# Prevalence and Related Factors of Anxiety Among University Teachers 1 Year After the COVID-19 Pandemic Outbreak in China: A Multicenter Study

**DOI:** 10.3389/fpsyt.2022.823480

**Published:** 2022-06-02

**Authors:** Wenning Fu, Xiaotong Han, Yifang Liu, Li Zou, Jing Wen, Shijiao Yan, Chuanzhu Lv

**Affiliations:** ^1^School of Nursing, Tongji Medical College, Huazhong University of Science and Technology, Wuhan, China; ^2^Department of Emergency Medicine, Hunan Provincial People's Hospital/The First Affiliated Hospital, Hunan Normal University, Changsha, China; ^3^Clinical Research Center for Emergency and Critical Care in Hunan Province, Changsha, China; ^4^School of Public Health, Hainan Medical University, Haikou, China; ^5^Emergency Medicine Center, Sichuan Provincial People's Hospital, University of Electronic Science and Technology of China, Chengdu, China; ^6^Key Laboratory of Emergency and Trauma of Ministry of Education, Hainan Medical University, Haikou, China; ^7^Department of Neurology, Zhongnan Hospital of Wuhan University, Wuhan, China

**Keywords:** COVID-19, university teachers, anxiety, China, mental health

## Abstract

**Objectives:**

This study aimed to evaluate the prevalence of anxiety among university teachers 1 year after the onset of the coronavirus disease 2019 (COVID-19) pandemic and provide empirical evidence of psychological intervention.

**Methods:**

A multicenter study was conducted to examine the prevalence of anxiety among 10,302 teachers in 21 Chinese universities from February 12 to April 23, 2021. The generalized Anxiety Disorder 7-item Scale (GAD-7) was used to assess symptoms of anxiety. Multivariate logistic regression models were used to analyze the relationship between potential influence and anxiety symptoms.

**Results:**

The overall prevalence of anxiety was 40.0% 1 year after the onset of the COVID-19 pandemic, which was found to be higher in women than in men (41.32% vs. 38.22%; *p* < 0.0001). The multivariate logistic regression showed that being the female (*OR* = 1.207; *95%CI*: 1.103–1.318), age ≥60 years (*OR* = 2.004; *95%CI*: 1.128–3.560), being married (*OR* = 1.319; *95%CI*: 1.150–1.513), and poor family economic status (*OR* = 1.580; *95%CI*: 1.321–1.891) were significantly associated with anxiety. Participants with moderate, slight, or no impact of COVID-19 on life (*OR* for moderate, 0.557; *95%CI*, 0.508–0.611; *OR* for slight/no, 0.377; *95%CI*, 0.323–0.439) showed a reduced risk of anxiety compared to those who reported a significant effect.

**Conclusions:**

Symptoms of anxiety were found in about two-fifths of Chinese university teachers 1 year after the outbreak of the COVID-19 pandemic. Our findings suggest that the government should improve the dynamic tracking of mental health and adopt long-term intervention strategies.

## Introduction

The Coronavirus disease 2019 (COVID-19) was first reported in China, becoming a global pandemic in March 2020 (1). However, it has not yet been completely controlled, even though it has been more than a year since the outbreak. COVID-19 and quarantine policies have spread anxiety throughout the population (2–4). According to the Global Burden of Disease Study (GBD) 2019, anxiety disorder is a disabling mental disorder and the leading cause of death (5). A previous study showed that approximately 34% of the general population reported moderate or above anxiety symptoms at the start of the pandemic in China (6).

Many countries adopted school closures as an effective measure to mitigate the spread of the pandemic (7). This accelerated shifts in educational approaches, leading to adverse effects on the mental health of teachers (8, 9). Prior to the COVID-19 pandemic, the prevalence of anxiety among teachers was only 4.98% in 2013 (10), which reached 13.67% in the first wave of the pandemic (February 4, 2020, to February 12, 2020) (11). As sociocultural populations, university teachers have borne the dual pressure of teaching and research and have been at a higher risk of psychological distress (10, 12). The spread of the pandemic might also change the psychological health status of university teachers. However, to the best of our knowledge, studies have not been conducted on the anxiety status of university teachers 1 year since the outbreak of the pandemic. Hence, this was the first and largest multicenter study to explore the prevalence of anxiety and related factors among teachers at 21 universities 1 year after the onset of the COVID-19 pandemic in China. Considering that humans would have to coexist with viruses for a long time, our study could provide clues for promoting the psychological health of teachers in this context.

## Methods

### Sample and Data Collection

This multicenter study was conducted 1 year after the outbreak of the COVID-19 pandemic worldwide (February 12–April 23, 2021). The study was approved by the Haikou Research Ethics Committee of Hainan Medical University. In this study, the structure of the questionnaire included a cover letter, instructions, questions and answers, and coding. Questionnaires for the online survey were sent put anonymously using the Questionnaire Star (https://www.wjx.cn). All respondents signed an electronic informed consent form before participating in the study. In addition, logic checks were built into the background system to ensure the quality and integrity of the study. The answers to all valid questionnaires were automatically entered into a data file and then checked by two independent researchers. Participants were not allowed to answer the questionnaire repeatedly, and each device (such as a mobile phone or computer) was only eligible for one response per question. The informed consent page presented two options (yes/no). Only participants who chose “yes” were taken to the questionnaire page. The questionnaire included questions about general demographic characteristics, concerns about COVID-19, the impact of COVID-19 on life, social support, and anxiety symptoms.

The formula for estimating the sample size of the survey rate is *n* = (*Z*_α/2_/δ)^2^ π (1- π). According to literature reports, the prevalence of anxiety among Chinese adults over the age of 18 is 4.98% (10), that is, π = 0.0498, *Z*_0.05/2_ = 1.96, α = 0.05, δ = 0.00498, then *N* = 7,330. Taking into account the invalid questionnaires, the sample size was set at 10,500. Based on the calculation results of sample size, in this study, the sampling process included two stages. In the first stage, 21 universities in Hainan Province were randomly selected based on a simple random sampling principle. In the second stage, online questionnaires were sent to the faculty and staff of the 21 selected universities through the Department of Academic Affairs and other departments. The inclusion criteria were participants who: (1) aged 18 years and older; (2) university teachers; (3) have provided informed consent electronically prior to registration. Exclusion criteria were participants who: (1) have been suffering from baseline psychological diseases; (2) offered the questionnaire with logical errors. Finally, 10,302 valid questionnaires were collected, with a response rate of 98.11%.

### Measurements

The Generalized Anxiety Disorder 7-Item Scale (GAD-7) was used to assess the degree of anxiety symptoms. The GAD-7 scale developed by Spitzer (13) was confirmed to have good factorial validity and reliability for the assessment of anxiety in the Chinese population (14). The scale contained seven items, with each item scored from 0 to 3, and the total scale score ranged from 0 to 21. According to the total score range, 0–4 points, 5–9 points, 10–14 points, and ≥15 points were considered as exhibiting no anxiety, mild anxiety, moderate anxiety, and severe anxiety, respectively. In the present study, the GAD-7 demonstrated high internal consistency (Cronbach's α = 0.94).

Social support was assessed using the Multidimensional Scale of Perceived Social Support (MSPSS) (15). The scale consisted of 12 items, with response options ranging from 1 (very strongly disagree) to 7 (very strongly agree). The MSPSS is used to assess the quality of social support from family, friends, and significant others in three categories. The scoring rule was as follows: the total scores ranged from 12 to 84, with higher scores representing higher levels of social support. The MSPSS scores of 12–36, 37–60, and 61–84 were considered to be low, medium, and high-level support, respectively. The MSPSS showed good factorial validity and reliability among teachers (16). Cronbach's alpha for the MSPSS was 0.97 in this study.

### Statistical Analysis

A descriptive analysis was conducted on the sociodemographic characteristics of teachers using frequency and percentage. The Chi-square test was used to compare demographic data, levels of social support, and prevalence of anxiety among the different groups. Additionally, multivariate logistic regression models were used to explore the influencing anxiety symptoms. All statistical analyses were performed using SPSS (version 21.0; SPSS Inc., Chicago, IL, USA). Furthermore, *p* < 0.05 (double-tailed) was considered statistically significant.

## Results

### General Sample Characteristics

A total of 10,302 university faculty and staff participated in the survey; of them 4,542 were men (44.09%) and 5,760 were women (55.91%). Most of them were aged 31–60 years (67.79%). In addition, some were aged ≤ 30 years (31.53%) and very few were aged ≥ 60 years (0.68%). In the level of social support, most perceived to have low support (71.11%), moderate support (27.74%), and high support (1.15%).

### The Prevalence and Differences of Anxiety Among University Teachers 1 Year After the COVID-19 Outbreak

The prevalence of anxiety was 40.0% 1 year after the COVID-19 pandemic, and it was higher in women than in men (41.32% vs. 38.22%, *p* < 0.05). Additionally, the prevalence of anxiety among those who reported a quite impact of COVID-19 on their lives was 49.16%. The distribution of anxiety symptoms in the population is not random and there are differences. There were statistically significant differences in the prevalence of anxiety among university teachers of different ages, working years, self-perceived family economic status, and social support (all *p* < 0.0001). The prevalence of anxiety among teachers who reported a greater impact of COVID-19 on life was significantly higher (*p* < 0.0001). In addition, marriage and occupation were associated with the prevalence of anxiety (*p* < 0.05) (Table 1).

**Table 1:** The anxiety of university teachers 1 year after COVID-19 pandemic.

**Variables**	**Total (*n*)**	**Anxiety (%)**	***F/t* value**	***P-*value**
**Gender**				
Male	4,542	1,736 (38.22)	10.1610	0.0014
Female	5,760	2,380 (41.32)		
**Age**				
18–30	3,248	1,403 (43.20)	34.0309	<0.0001
31–40	3,653	1,473 (40.32)		
41–50	2,301	863 (37.51)		
51–60	1,030	350 (33.98)		
>60	70	27 (38.57)		
**Ethnic group**				
Ethnic Han	9,379	3,750 (39.98)	0.0381	0.8453
Others	923	366 (39.65)		
**Years of work**				
1–5	3,853	1,662 (43.14)	47.3024	<0.0001
6–10	1,923	785 (40.82)		
11–20	2,582	1,007 (39.00)		
21–30	1,278	452 (35.37)		
>30	666	210 (31.53)		
**Marriage**				
Not-married	3,173	1,311 (41.32)	11.0638	0.0114
Married	6,761	2,681 (39.65)		
Widowed	40	9 (22.50)		
Divorced	328	115 (35.06)		
**Self-perceived family economic status**				
Good	1,053	330 (31.34)	149.1549	<0.0001
Fair	7,544	2,894 (38.36)		
Bad	1,705	892 (52.32)		
**Impact of COVID-19 on life**				
Quite impacted	5,350	2,630 (49.16)	431.9809	<0.0001
Moderately impacted	3,752	1,218 (32.46)		
Slightly or not impacted	1,200	268 (22.33)		
**Concern about COVID-19**				
Quite concerned	9,615	3,844 (39.98)	0.0067	0.9347
Moderately concerned	663	261 (39.37)		
Slightly or not concerned	24	11 (45.83)		
**Social support**				
High	118	59 (50.00)	286.2510	<0.0001
Moderate	2,858	1,511 (52.87)		
Low	7,326	2,546 (34.75)		
**Total**	10,302	4,116 (39.95)		

### The Influential Factors Associated With Anxiety University Teachers 1 Year After the COVID-19 Outbreak

Screening positive for anxiety among university teachers was associated with being female, age >60 years, married, bad family economic status, 1–5 years of work, and a quite impact of COVID-19 on life. The multivariate logistic regression analysis showed that female teachers had a higher risk of anxiety symptoms (*OR* = 1.207; 95%*CI*: 1.106–1.318). Compared with teachers aged ≤ 30 years, those aged ≥60 years had a significantly higher risk of anxiety (*OR* = 2.004; 95%*CI*: 1.128–3.560). Additionally, there was a higher risk of anxiety in married teachers (*OR* = 1.319; 95%*CI*: 1.150–1.513) than in unmarried teachers. In addition, those who reported poor family economic status were associated with a higher risk of anxiety than those who reported good economic status (*OR* = 1.580; 95%*CI*: 1.321–1.891). However, teachers who had worked 11–20, 20–30 years, and longer than 30 years showed a lower risk of anxiety than teachers who had worked for 1–5 years. Those who reported a moderate, slight, or no impact of COVID-19 on their lives showed a reduced risk of anxiety compared to those who reported a quite impact (*OR* for moderate, 0.557; 95%*CI*, 0.508–0.611; *OR* for slight/no, 0.377; 95%*CI*, 0.323–0.439) (Table 2).

**Table 2:** Multivariate logistic regression analysis of factors associated with university anxiety among university teachers.

***Variables***		***SE***	***Wald***	***P***	***OR***	***95%CI***
**Gender**						
Male	Reference					
Female		0.0447	17.6962	<0.0001	1.207	1.106–1.318
**Age**						
18–30	Reference					
31–40		0.0790	0.6344	0.4258	0.939	0.804–1.096
41–50		0.0982	0.1142	0.7354	1.034	0.853 1.253
51–60		0.1333	1.1396	0.2857	1.153	0.888–1.497
>60		0.2933	5.6145	0.0178	2.004	1.128–3.560
**Years of work**						
1–5	Reference					
6–10		0.0723	2.0983	0.1475	0.901	0.782–1.038
11–20		0.0764	3.8941	0.0485	0.860	0.740–0.999
21–30		0.1027	4.0097	0.0452	0.814	0.666–0.996
>30		0.1478	7.5007	0.0062	0.667	0.499–0.891
**Marriage**						
Not-married	Reference					
Married		0.0699	15.7075	<0.0001	1.319	1.150–1.513
Widowed		0.4025	3.0645	0.0800	0.494	0.225–1.088
Divorced		0.1412	0.0469	0.8286	1.031	0.782–1.360
**Self-perceived family economic status**						
Good	Reference					
Fair		0.0761	2.9402	0.0864	1.139	0.982–1.323
Bad		0.0915	25.0190	<0.0001	1.580	1.321–1.891
**Impact of COVID-19 on life**						
Quite impacted	Reference					
Moderately impacted		0.0473	153.0590	<0.0001	0.557	0.508–0.611
Slightly or not impacted		0.0787	154.0149	<0.0001	0.377	0.323–0.439
**Social support**						
High	Reference					
Moderate		0.1985	3.0424	0.0811	1.414	0.958–2.086
Low		0.1965	2.9217	0.0874	0.715	0.486–1.050

## Discussion

This multicenter study investigated anxiety symptoms among 10,320 teachers from 21 universities 1 year after the start of the COVID-19 pandemic. The results indicated that a significant proportion of the university faculty and staff had mental health problems, with 4,542 (40.0%) participants reporting anxiety symptoms. Previous studies confirmed that the prevalence of anxiety increased owing to COVID-19 (17, 18). The percentage of anxiety among university teachers in this study is close to 34.6% of that reported in a survey of university professors when the COVID-19 pandemic outbreak almost 1 year in Brazil (19). That is, anxiety symptoms seem to be very common among university teachers during the COVID-19. University teachers undertake the task of teaching and play the role of researchers (20). Owing to the COVID-19 pandemic, many university teachers could not continue their research projects. A study of teachers from kindergarten to university in China in the same period showed that 17.7% of teachers reported symptoms of anxiety, with a significantly higher percentage of university teachers reporting moderate and severe anxiety than teachers in other types of schools (21). Therefore, we suggest that the COVID-19 is a more significant psychological challenge for university teachers. Studies have shown that negative psychological emotions, such as stress and anxiety, have an impact on teachers' health (11, 22), leading to a decrease in their work enthusiasm and a decline in teaching quality (23). Simultaneously, anxiety is also an important cause of death among teachers (24). Therefore, a comprehensive investigation and intervention should be conducted on the mental health of university teachers in the current pandemic situation.

We also found that gender, age, marriage, economic status, years of work, and the degree of impact of COVID-19 on life were associated with anxiety. As in previous studies, women have been identified to be at a higher risk of mental health problems (11, 25). We believed that the possible mechanisms involved physical and psychological components. Influenced by gender chromosome genes and psychological characteristics, women are found to exhibit more self-blame in stressful events and show a tendency toward avoidance, depression, and other negative coping methods, which are closely related to the increase in anxiety symptoms in women (26). Additionally, we found that participants aged ≥60 years were more likely to have anxiety than those aged ≤ 30 years. First, older teachers had a higher risk of infection and poorer prognosis. Consequently, health stress and negative emotions were worse in older than in younger people, as confirmed in other studies (27, 28). Second, a recent study has confirmed that social networks could influence mental health in older adults who have struggled to reap the benefits of electronic social networks. COVID-19 has resulted in prolonged social isolation among older individuals, leading to aggravated anxiety symptoms (29). Interestingly, we found that the risk of anxiety among married university teachers was 1.319 times higher than that of unmarried teachers. Previous research also showed that married teachers appear to be under greater stress. They are required to take on more family responsibilities and worry more about parents and children influence the COVID-19 than unmarried teachers (22, 30). In addition, studies have shown that COVID-19 exacerbates teachers' job instability and increases the rate of layoffs, thus increasing the economic pressure on teachers (25, 31). This phenomenon was also reflected in our study in that teachers with poor economic status had a higher detection rate of anxiety symptoms. Furthermore, the risk of anxiety was higher among teachers with <5 years of experience. The reasons for this may be attributed to the fact that new teachers who graduate from college and enter the workforce with low control over the content of their work (32). According to previous studies, teachers with more years of experience are more capable of solving problems independently in their daily work (33). Therefore, they have a higher ability to cope with the dual stress of the pandemic and the profession. Even though, there were differences in the risk of anxiety among university teachers in different occupation types, occupation type was not an influential factor in teacher anxiety. Thus, all teachers should be covered, whether they are in teaching positions, management positions, or others, when adopting psychological interventions for university teachers. The results of this study showed that the degree of impact of COVID-19 on life was an important influencing factor for university teachers. This is in line with a study conducted by Fu (4). Evidently, individuals whose lives are severely impacted by COVID-19, especially those who have lost family members, should be the focus of our subsequent intervention.

The relationship between social support and mental health was not conclusive for a long time (34, 35). Many scholars generally regarded social support as a protective factor for mental health; a lower level of social support is negatively correlated with anxiety symptoms (34). However, the findings of this study revealed a different viewpoint. This may be attributed to the following reasons: (1) the protective effect of perceived social support on university teachers was weak; and (2) the number of teachers with a high level of perceived social support was very small in this study, and more than half of the teachers had a low level of social support; therefore, the sample size should be increased to confirm the accuracy of the research conclusion. Nevertheless, further expansion of our study is needed to assess the stability and reliability of our results.

### Strengths and Limitations

This study had several advantages. First, to the best of our knowledge, this is the first and largest multicenter survey of anxiety among university teachers conducted 1 year after the outbreak of the COVID-19 pandemic. Second, this study showed that nearly half of the university teachers had psychological problems. Considering the continued spread of the pandemic and the complexity of its psychological impact on teachers, this study could provide a valuable reference for the management of psychological problems among teachers in other regions and countries. However, our study had a limitation, in that it was a cross-sectional study and lacked longitudinal follow-up. Therefore, causality could not be established. Hence, further investigation is required on the long-term psychological effects of the pandemic on teachers. In addition, the universities included in this study were all public institutions, and the data collection did not collect information on teachers' anxiety at different levels, and there were some limitations in extrapolating the research results to different levels of teachers in more public and private universities.

## Conclusion

About two-fifths of Chinese university teachers experienced anxiety symptoms 1 year after the start of the COVID-19 pandemic. Therefore, the government should focus on the mental health of teachers, particularly female and older teachers. In addition, we believed that dynamic and long-term psychological intervention measures should be taken to reduce the adverse psychological effects of the COVID-19 pandemic on teachers. These findings might be useful for providing a current anxiety profile of university teachers 1 year after the onset of the COVID-19 and pandemic for functioning as a reference point for further studies.

## Data Availability Statement

The original contributions presented in the study are included in the article/supplementary materials, further inquiries can be directed to the corresponding author/s.

## Ethics Statement

The studies involving human participants were reviewed and approved by Research Ethics Committee of Hainan Medical University in Haikou, China. The patients/participants provided their written informed consent to participate in this study.

## Author Contributions

WF, YL, and CL conceived and designed the study. LZ, SY, JW, XH, and WF participated in the acquisition of data. WF, XH, and SY analyzed the data. YL, XH, and CL gave advice on methodology. WF drafted the manuscript, XH, YL, and CL revised the manuscript. All authors read and approved the final manuscript.

## Funding

This study was funded by the Hainan Provincial Science and Technology Major Project (ZDKJ202004), the National Natural Science Foundation of China (Grant. 72104082), the Program of Excellent Doctoral (Postdoctoral) of Zhongnan Hospital of Wuhan University (Grant. ZNYB2021003), and the Key Laboratory of Emergency and Trauma (Hainan Medical University), Ministry of Education (Grant. KLET-202002).

## Conflict of Interest

The authors declare that the research was conducted in the absence of any commercial or financial relationships that could be construed as a potential conflict of interest.

## Publisher's Note

All claims expressed in this article are solely those of the authors and do not necessarily represent those of their affiliated organizations, or those of the publisher, the editors and the reviewers. Any product that may be evaluated in this article, or claim that may be made by its manufacturer, is not guaranteed or endorsed by the publisher.
